# A Survey of Regulatory Interactions Among RNA Binding Proteins and MicroRNAs in Cancer

**DOI:** 10.3389/fgene.2020.515094

**Published:** 2020-09-08

**Authors:** Ying Liu, Chu Pan, Dehan Kong, Jiawei Luo, Zhaolei Zhang

**Affiliations:** ^1^College of Computer Science and Electronic Engineering, Hunan University, Changsha, China; ^2^Donnelly Centre for Cellular and Biomolecular Research, University of Toronto, Toronto, ON, Canada; ^3^Department of Statistical Sciences, University of Toronto, Toronto, ON, Canada; ^4^Department of Computer Science, University of Toronto, Toronto, ON, Canada; ^5^Department of Molecular Genetics, University of Toronto, Toronto, ON, Canada

**Keywords:** RNA binding proteins, microRNAs, gene regulation, gene expression, cancer, regression analysis

## Abstract

Recent advances in genomics and proteomics generated a large amount of *trans* regulatory data such as those mediated by RNA binding proteins (RBPs) and microRNAs. Since many *trans* regulators target 3′ UTR of mRNA transcripts, it is likely that there would be interactions, i.e., competitive or cooperative effect, among these *trans* factors. We compiled the available RBP and microRNA binding sites, mapped them to the mRNA transcripts, and correlated the binding data with mRNA expression data generated by The Cancer Genome Atlas (TCGA). We separated pairs of RBPs and microRNAs into three scenarios: those that have overlapping target sites on the same mRNA transcript (*overlapping*), those that have target sites on the same mRNA transcript but non-overlapping (*neighboring*), and those that do not target the same mRNA transcript (*independent*). Through a regression analysis on expression profiles, we indeed observed interaction effect between RBPs and microRNAs in the majority of the cancer expression data sets. We further discussed implication of such widespread interactions in the context of cancer and diseases.

## Introduction

Post-transcriptional regulation is an important yet complex system that modulates expression level of genes inside cells. The commonly studied post-transcriptional regulatory mechanisms include alternative splicing, mRNA modification, regulation by RNA binding proteins (RBP) or by microRNAs. Dysregulation in post-transcriptional regulation has been implicated in many human diseases, especially those mediated by RBPs (Lukong et al., 2008) and by microRNAs (Calin and Croce, 2006). Since many RBPs and microRNAs target the 3′ UTR of the same mRNA transcripts, it is likely that there exist competitive or cooperative interactions among these *trans* regulators. For example, these *trans* regulators could compete for the same binding sites on the 3′ UTR; alternatively, binding by one *trans* regulator could affect the local mRNA conformation and influence binding by other *trans* regulators. Indeed, a number of earlier studies have investigated interactions between these regulators. Perhaps the most widely studied is the competition between microRNAs, either via competing for the same AGO protein complex or via indirectly modulating the abundance of mRNAs. Califano, Pandolfi, and colleagues proposed the idea that different types of RNAs, either mRNAs or non-coding RNAs, can compete for the same microRNAs, and changes in RNA abundance can modulate the regulatory effect of other microRNAs, hence the concept of ceRNA (competing endogenous RNAs) (Salmena et al., 2011; Sumazin et al., 2011; Li et al., 2014; Tay et al., 2014). Marks and colleagues observed that overexpression of one type of microRNA can overload the AGO proteins thus reduce the possibility of other microRNAs being loaded onto the RISC complex (Khan et al., 2009). In addition to these computational analyses, there are experimental evidence supporting such competitive effects among microRNAs (Hua et al., 2006; Castanotto et al., 2007; Ebert et al., 2007).

Doyle and Tenenbaum (2014) presented a general framework on competition among RBPs; however, there have not been any large-scale experimental survey in this area except for a few cases involving HuR. It was shown that, HuR and AUF1, two RBPs that cause opposing effect on their targets (the binding of HuR stabilizes mRNA while binding of AUF1 promotes degradation), compete for common target sequences and can modulate each other’s effect (Barker et al., 2012). Competition between HuR and wild-type TTP for binding to the HuR transcript has been implicated in HuR regulation and its cytoplasmic localization (Al-Ahmadi et al., 2009). HuR is a widely studied RBP and binds to many target mRNAs, so it is not surprising that many of the reported competition events involved HuR. Zarnack et al. (2013) conducted genome-wide iCLIP experiments and discovered that HNRNP (Heterogeneous Nuclear Ribonucleoprotein C) competes with the splicing factor U2AF65 at many splice sites, which prevents the exonization of *Alu* elements and protects the integrity of human transcriptome. In another example, it was found that two families of splicing factors, CELF and RBFOX, can antagonize each other in the regulation of global splicing events (Gazzara et al., 2017). Regarding interactions between RBPs and microRNAs, Srikantan et al. (2012) presented an overview of potential competition and cooperation between HuR and microRNAs. For example, it was shown that HuR can recruit miR-19 and the associated RISC complex to the mRNA transcript of small GTPase RhoB and repress protein translation; the interaction between HuR and miR-19 can be decoupled by UV exposure, which prevents the down-regulation of RhoB by miR-19 (Glorian et al., 2011). Such regulatory role of HuR has later been reported for let-7, miR-17-92 and other microRNAs as well (Kim et al., 2009; Kundu et al., 2012; Gunzburg et al., 2015; Mihailovich et al., 2015).

**FIGURE 1:**
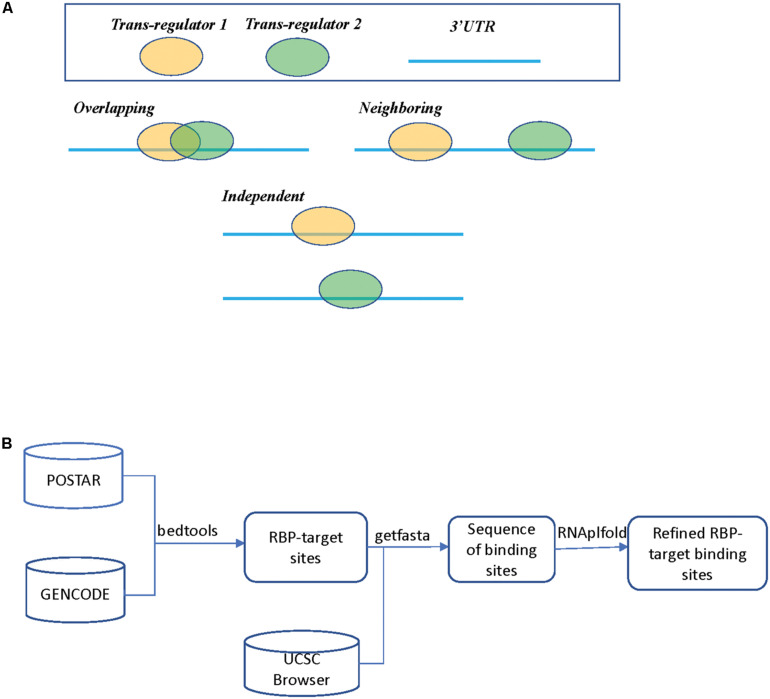
**(A)** Three different scenarios between two *trans* regulators. In scenario 1 (*Overlapping*), both *trans* regulators target the same mRNA transcript with overlapping binding sites. In scenario 2 (*Neighboring*), both *trans* regulators target the same mRNA transcripts but the binding sites do not overlap. In scenario 3 (*Independent*), the two *trans* regulators do not bind to the same transcript. **(B)** A flowchart on determining RBP binding sites.

Despite these published studies, to the best of our knowledge, there still lacks a general analysis of global regulatory interactions between RBPs and microRNAs beyond the well-studied proteins such as HuR. In this study, we limited our list of RBPs only to those that target 3′ UTR of mRNAs and excluded those binding events at the 5′ UTR or coding regions (CDS). We also did not include splicing factors that bind to intron and exon junctions, which warrants a separate study. For RBP binding sites, we collected and mapped the binding sites derived from published experimental studies, specifically the POSTAR database (Hu et al., 2017). These RBP binding sites were further refined by considering RNA accessibility to include only those deemed of high confidence. The microRNA target sites were derived from computational predicted databases. Based on the binding sites of RBPs and microRNAs, Figure 1A shows three possible scenarios, which we refer to as “*Overlapping*,” “*Neighboring*,” and “*Independent*,” respectively.

## Materials and Methods

### Mapping MicroRNA and RBP Target Sites

The microRNA targeting information was downloaded from TargetScan database (version 7.2) (Agarwal et al., 2015), which as of January 2019 contained 321 human microRNAs and 11,354 target genes. We chose to use TargetScan since it was more frequently updated (last updated in March 2018). The default parameters for target prediction were used. We did not limit our study to only experimentally validated targets such as those curated by TarBase (Karagkouni et al., 2018), since we aimed to finding global competitive effect between *trans* factors, rather than investigating individual factors. The RBP binding sites were downloaded from POSTAR2^1^ (Hu et al., 2017), the coordinates of these binding sites were based on GENCODE Human release 27. The coordinates of microRNA and RBP binding sites were mapped from hg19 to hg38 by using the LiftOver tool. The definition of 3′ UTR from GENCODE was used. Details on the further mapping and processing of these target sites are described in Results.

### Randomization of RBP and MicroRNA Target Sites

We conducted randomization on the *overlapping* RBP (or microRNA) target sites to calculate the baseline probability for two RBPs to have overlapping target sites on an mRNA transcript, provided that they already have target sites on the same mRNA transcript. For a given mRNA, we randomly re-distributed RBP target sites on the 3′ UTR region and compared the number of overlapping RBP target sites with the real data. We conducted randomization 10 times and took the average number of overlapping target sites.

### TCGA Datasets

The mRNA and microRNA expression data were downloaded from Broad Institute Firehose website^2^. Genes that have missing value among over 30% of the samples were not considered. We used the *knn.impute* package to fill in the missing value. The final summary of The Cancer Genome Atlas (TCGA) data is shown in Supplementary Table S1.

### Statistical Analysis

We conducted regression analysis as formulated in Eq. 1 to measure the significance of interactions among *trans* regulators. We used the R package p.adjust to calculate the *p*-values adjusted by Benjamini–Hochberg method (*q*-value < 0.05). The *p*-value for each term tests the null hypothesis that the coefficient is equal to zero (no effect). A low *p*-value (<0.05) indicates that there is a larger interaction probability between the two *trans* regulators. We used the R function ks.test (*x*, *y*, “*greater*”) to carry out the Kolmogorov–Smirnov test; *x* and *y* represent the *p*-values calculated from Eq. 1 from *Overlapping*, *Neighboring*, and *Independent* regulator pairs. The “*greater*” option tests the null hypothesis that *x* is stochastically smaller than *y*.

## Results

### Mapping RBP Targets

The analysis flowchart is shown in Figure 1B and details on how we derived RBP and microRNA binding sites can be found in the section “Materials and Methods.” Supplementary Table S2 summarizes the downloaded RBP binding data. The POSTAR2 database had collected global binding data for 171 RBPs (as of January 2019), mostly were determined by CLIP-Seq or similar approaches and analyzed by software such as PARalyzer or CIMS (Corcoran et al., 2011; Zhang and Darnell, 2011). We noticed that there was often more than one study on a unique RBP; we pooled the binding sites determined from these separate studies. For those genes with multiple alternative mRNA transcripts, we used GENCODE as a reference and selected the transcript with the longest 3′ UTR. Many of these RBP binding sites are likely only bound by RBP in specific cell types due to cell type specific abundance of the RBPs and the target mRNAs, or the competitive or cooperative interaction between RBPs. At this step in our study, we simply mapped all the potential RBP binding sites and their spatial relationship in preparation for the subsequent regression analysis on expression levels.

We noticed that many of the RBP binding sites reported by the PARalyzer or eCLIP were very long and spanned over 50 nucleotides, which was characteristic of the broad peaks called by peak calling software (Van Nostrand et al., 2016) or was the result of multiple overlapping binding sites being merged together (Corcoran et al., 2011). We manually examined the annotation of these RBPs and the RNA binding domains (RBD) within these proteins; the majority of these RBPs or RBDs had evidence to bind to single stranded RNAs (ssRNA) instead of double stranded RNAs (dsRNA). Therefore, we further refined these RBP binding sites by calculating the local RNA structure accessibility and only retained those that were considered to be accessible to RBPs. We used the RNAplfold program in the Vienna Package and used recommended parameters (*W* = 80 and *L* = 40) (Lorenz et al., 2011). It was previously shown that inclusion of local RNA accessibly could help improve the identification of RBP binding events (Tafer et al., 2008).

After these filtering steps, we derived a final RBP target set, which included 171 distinct RBPs, 14,520 mRNA transcripts, and 2,865,656 target sites. On average, after considering RNA accessibility, a typical RBP was found to bind 2,077 genes (median 502 genes), and on average each gene was targeted by 24.5 RBPs (median 23 RBPs) (Table 1). We found that a few proteins such as HNRNPC and DDX3X had the most targets (11 RBPs had more than 8000 targets each). These RBPs are among the most studied proteins and have the greatest number of CLIP-Seq experiments done on them. On the other hand, a gene can be targeted by up to 92 RBPs (e.g., HNRNPA2B1 and MAFG, Table 2). The detailed data can be found in Supplementary Tables S3, S4.

**TABLE 1:** Top 10 RBPs that have the highest number of target genes and target binding sites as annotated by POSTAR2, sorted by the number of target genes **(A)** and number of total binding sites **(B)**, after considering RNA accessibility.

RBP name	Number of target genes (pooled over multiple studies)	Number of binding sites (pooled over multiple studies)	Average number of binding sites per target gene
**(A) Sorted by number of target genes**			
AXTN2	11,286	229,709	20.4
ELAVL1 (HuR)	9,746	193,408	19.8
HNRNPC	8,644	190,668	22.1
TARDBP (TDP-43)	10,526	154,190	14.6
LIN28B	10,185	112,430	11.0
MOV10	7,963	111,789	14.0
DDX3X	9,426	101,424	10.8
UPF1	8,077	99,391	12.3
ZC3H7B	6,807	98,351	14.4
CPSF7	8,154	97,348	11.9

**(B) Sorted by number of total binding sites**			

ATXN2	11,286	229,709	20.4
TARDBP (TDP-43)	10,526	154,190	14.6
LIN28B	10,185	112,430	11.0
ELAVL1 (HuR)	9,746	193,408	19.8
DDX3X	9,426	101,424	10.8
FIP1L1	8,929	59,958	6.7
CSTF2T	8,919	86,762	9.7
NUDT21	8,913	49,380	5.5
HNRNPC	8,644	190,668	22.0
CPSF7	8,154	97,348	11.9

**TABLE 2:** Human genes that have the highest number of RBP regulators.

Gene name	Number of unique RBP regulators	Number of pooled RBP binding sites	Number of binding sites that overlap with another RBP binding site, another miRNA binding site, or another RBP or miRNA binding site
**(A) Sorted by the number of total RBP binding sites**			
CBX5	75	4827	4789/329/4794
NUCKS1	88	4324	4311/365/4311
NUFIP2	74	4015	3974/559/3976
PANK3	79	3720	3674/201/3678
ZNF207	80	3564	3484/267/3487
C16orf72	72	3381	3333/NA/3333
AGO2	85	3354	3277/62/3279
G3BP1	77	3225	3174/209/3180
CDK6	78	2903	2840/172/2844
SCD	80	2722	2711/86/2713

**(B) Sorted by the number of unique RBP regulators**			

HNRNPA2B1	92	1687	1679/163/1679
MAFG	92	1166	1137/74/1141
NUCKS1	88	4324	4311/365/4311
SRSF1	88	2199	2197/360/2197
MAZ	86	1028	1024/69/1024
AGO2	85	3354	3277/62/3279
PDXK	85	1307	1257/32/1257
DNAJC5	84	1049	1016/63/1016
LENG8	84	1559	1549/30/1549
FOXK1	83	2068	2002/63/2003

***(A)** Sorted by the number of total RBP binding sites, **(B)** sorted by the number of unique RBP regulators.*

### Overlap Among RBP Binding Sites

As shown in Figure 1A, we separated pairs of *trans* factors into three scenarios: *Overlapping*, *Neighboring*, and *Independent*. As described above, in this study we did not consider alternative splicing or alternative 3′ UTRs; instead we choose the longest 3′ UTR for each human gene. We define two *trans* regulators having *overlapping* target sites if their target sites, either experimentally determined or predicted, overlap by at least 1 nucleotide. We experimented with alternative definition such as defining overlapping as distance on the mRNA being less than 5 nucleotides, such alterations had no effect on the final conclusions.

**FIGURE 2:**
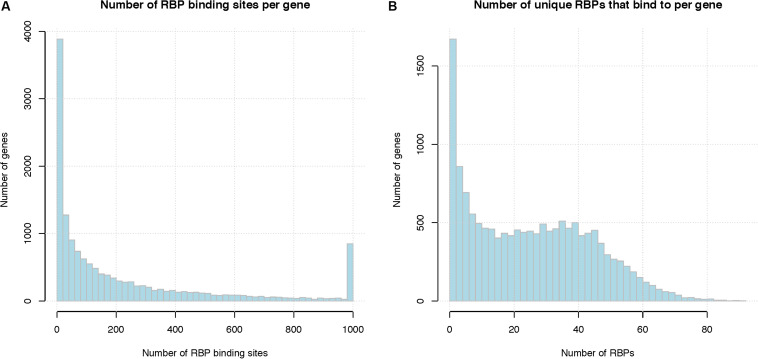
Summary of RBP binding sites. **(A)** Number of RBP binding sites per gene, binding sites from different RBPs are pooled together. *X*-axis represents the number of RBP binding sites per gene and the last column represents the number of genes with total binding sites greater than 1000, *Y*-axis indicates the number of genes that have that number of RBP binding sites. **(B)** Number of unique RBPs that bind to a human gene. *X*-axis indicate the number of unique RBPs bound to one mRNA, *Y*-axis indicates the number of genes.

Figure 2A shows the distribution of the number of RBP binding sites per gene, after pooling binding sites from different RBPs for the same gene. On average, a human mRNA transcript has 197 RBP binding sites (median 85 binding sites). Figure 2B shows the number of distinct RBPs that target each mRNA. On average, a human mRNA transcript is targeted by 24.5 distinct RBPs (median 23 RBPs). We note that these statistics are based only on the 171 RBPs for which there are *in vivo* binding data; the number of RBPs that target each mRNA is likely higher.

**FIGURE 3:**
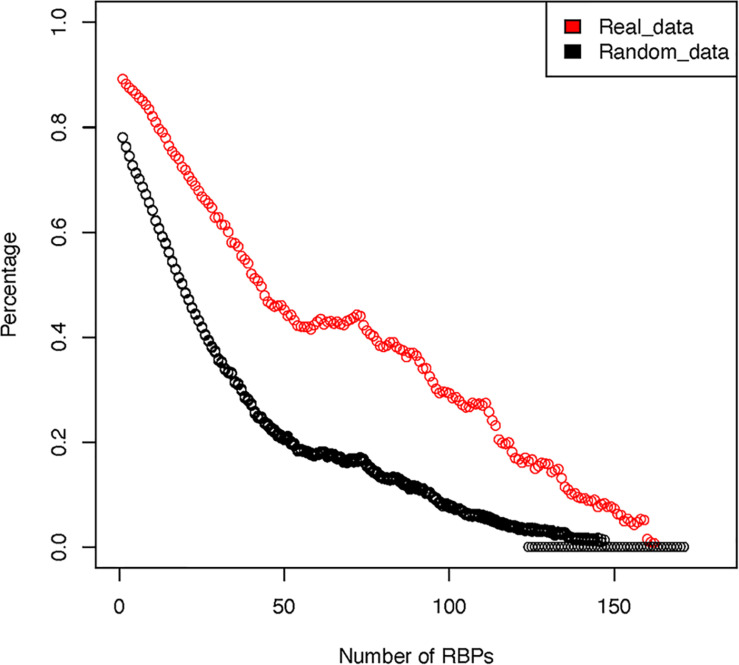
Overlap among RBP binding sites after sequentially removing RBPs with the highest number of binding sites. The red circles represent real observed data while the black circles represent averaged results over 10 rounds of random simulation.

For the 171 RBPs, our analysis show that they have a total of 2,865,656 potential binding sites against the entire human transcriptome. Among these binding sites, 2,579,262 (90%) overlap with another RBP binding site. In contrast, in our random simulations, approximately 2,236,200 (78.1%) of the binding sites overlap with another binding site. We next tested whether such high degree of overlap was caused by the very few RBPs that have many binding sites throughout the transcriptome. To test such possibility, we sequentially removed the RBPs that had the highest number of binding sites and re-calculated the frequency of overlap (Figure 3). The results showed that, even after removing the top 5 RBPs that have the most abundant target sites, on average, 86.3% of the RBPs still have overlapping sites with another RBP (70.1% in random simulations). This indicates that indeed many RBPs share potential overlapping binding sites, at least as determined by pull-down experiments. Similar observation had been made by other authors from the study of *in vitro* determined binding affinities of RBPs (Van Nostrand et al., 2016). There are two potential explanations for the observed significant overlap between the potential RBP binding sites. A mechanistic explanation is that RBPs preferably recognize RNA regions that have unique nucleotide composition, favorable structural conformation and accessibility. Many of these RBPs share homologous RNA binding domains (RBD), thus it is likely that they bind to similar target sequence motifs. Alternatively, such overlap could be the result of millions of years of adaptive evolution and selection.

**FIGURE 4:**
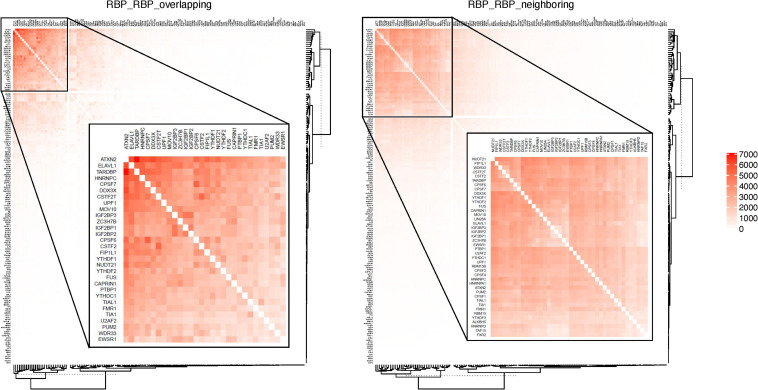
RBPs that have overlapping or neighboring binding sites. Color in each cell indicates the number of mRNAs on which the two RBPs have *overlapping* or *neighboring* binding sites. RBPs are listed in the same order on both *X*- and *Y*-axis. The top overlapping pairs in the top left corer are magnified in the inset.

Table 2 shows some of the genes that are bound by the greatest number of RBPs, also shown are the number of binding sites, and the number of binding sites that overlap with another binding site. The complete list of such data is shown in Supplementary Table S5. Table 3 lists the RBP pairs that have the highest number of overlapping binding sites. HNRNPC, ELAVL1 (HuR), TARDBP (TDP-43), and ATXN2 are among the RBPs that have the highest number of binding sites and target genes (see Table 1); they also have the highest number of overlapping binding sites and overlapping mRNA targets. Figure 4 shows the network of *overlapping* and *neighboring* RBPs, represented as a heatmap, detailed data are in Supplementary Table S6.

**TABLE 3:** List of RBP-RBP pairs that have the highest number of *overlapping* and *neighboring* binding sites.

RBP 1	RBP 2	Number of *overlapping* binding sites	Number of target genes with *overlapping* binding sites	Number of target genes with *neighboring* binding sites
HNRNPC	ELAVL1 (HuR)	73,554	5346	2379
TARDBP (TDP-43)	ATXN2	72,801	6938	2707
ELAVL1 (HuR)	ATXN2	70,707	5955	2851
HNRNPC	ATXN2	67,260	5759	2372
TARDBP (TDP-43)	HNRNPC	53,022	5371	2509
ZC3H7B	ELAVL1 (HuR)	49,607	4801	1709
TARDBP (TDP-43)	ELAVL1 (HuR)	49,519	5299	3270
CPSF7	ATXN2	48,239	5641	2194
MOV10	ATXN2	48,026	5049	2610
LIN28B	ELAVL1 (HuR)	47,457	6521	2028

*The first two columns are the names of the RBPs, the 3rd column is the number of *overlapping* binding sites between these two RBPs, the 4th column is the number of genes on which these two RBPs have *overlapping* binding sites, the 5th column is the number of genes on which these two RBPs have *neighboring* binding sites.*

Figure S1 show that, at least as suggested by sequence data, there exists prevalent potential overlap among RBP binding sites. For any RBP-RBP pair, there is 80.1% chance (74% in randomized simulation) that they have *overlapping* binding sites on at least one mRNA target; and 94.6% chance that they have *neighboring* binding sites on at least one mRNA. There is 19% chance (15.2% in randomized simulation) that any pair of RBPs have *overlapping* binding sites on at least 100 mRNAs, and 38% chance that they have *neighboring* binding sites on at least 100 mRNAs.

### Mapping MicroRNA Target Sites

**FIGURE 5:**
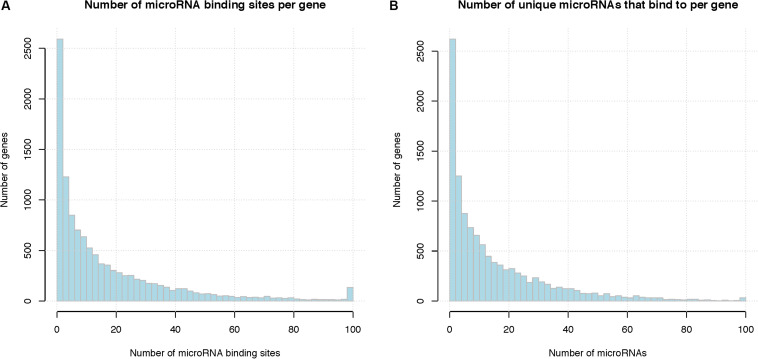
Summary of microRNA binding sites. **(A)** Total number of microRNA binding sites per gene, binding sites of different microRNAs are pooled together. *X*-axis represents the number of microRNA binding sites per gene and the last column represents the number of genes with binding sites greater than 100, *Y*-axis indicates the number of genes that have that number of microRNA binding sites. **(B)** Number of unique microRNAs that bind to a human gene. *X*-axis represents the number of microRNA binding sites per gene and the last column represents the number of genes with binding sites greater than 100, *Y*-axis indicates the number of genes.

On average, each microRNA is predicted to regulate 557 genes (median 433 genes), and each gene is predicted to be targeted by 16 microRNAs (median 9 microRNAs). Figure 5 shows the distribution of microRNA binding sites among the genes; more detailed information can be found in Supplementary Tables S7, S8. Among the total of 201,235 microRNA binding sites, 157,245 (78%) overlap with another microRNA binding site. There are several previously published studies on overlap and interaction between microRNA regulations including those from our own group (Li et al., 2014), therefore, we focused on interactions between RBPs and microRNAs in this study. Figure S2 shows the overlap between microRNA regulations, represented as a heatmap. Supplementary Table S9 lists the microRNA pairs that have the highest number of *overlapping* binding sites. Since these top microRNA pairs often belong to the same family and are highly similar in sequence, their binding sites are almost always deemed as *overlapping*, i.e., no *neighboring* binding sites between these pairs. The complete list is shown in Supplementary Table S10. It should be noted that human microRNAs consist of many families of homologous microRNAs that share similar seed sequences, e.g., hsa-miR-15a and has-miR-15b; these microRNAs would naturally have *overlapping* binding sites. We treated these homologous microRNAs as independent regulatory elements since they do not always have similar expression levels across cell types.

### Overlap Between RBP and MicroRNA Binding Sites

We next investigated overlap between RBP and microRNA binding sites. As described above, our final dataset included a total of 2,865,656 RBP binding sites mediated by 171 RBPs, and 201,235 microRNA binding sites mediated by 321 microRNAs. On average, each mRNA transcript had 197 RBP binding sites (median 85) and 18 microRNA binding sites (median 9). Among the 2,865,656 RBP binding sites, 181,690 (6.3%) (average 243,260, or 8.5% in randomized simulations) overlapped with another microRNA binding site, while 2,361,587 (82.4%) had *neighboring* microRNA binding sites on the same mRNA transcript. It is surprising that in the randomized simulations more RBP binding sites overlap with another microRNA binding site; one possible explanation is that the real RBP binding sites tend to cluster together in the 3′ UTR thus the randomized RBP binding sites had more chance to overlap with randomized microRNA binding sites. Among the total of 201,235 microRNA binding sites, 111,300 (55.3%) (average 109,503, 54% in randomized simulations) overlapped with an RBP binding site, 11,371 (5.7%) had a *neighboring* RBP binding site. These results including the simulation results suggests potential prevalent interactions between RBPs and microRNAs.

**FIGURE 6:**
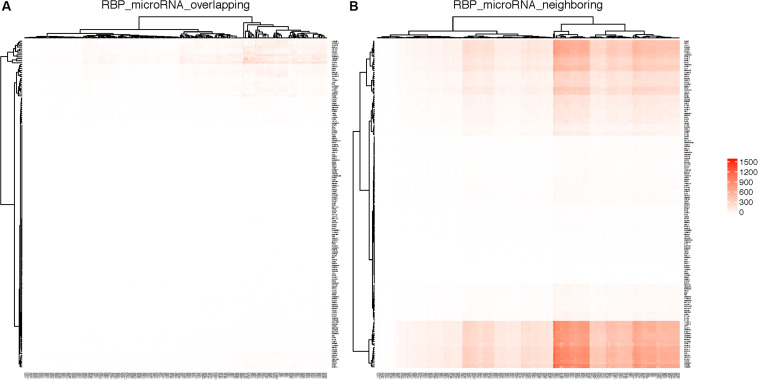
RBPs and microRNAs that have overlapping or neighboring binding sites. RBPs are listed on the *Y*-axis and microRNAs are listed on the *X*-axis. Color in each cell represents the number of genes on which the RBP and the microRNA have *overlapping* **(A)** or *neighboring* **(B)** binding sites. As the result of clustering, the order of RBPs or microRNAs are different between left and right panels.

Figure 6A shows the RBPs and microRNAs that have *overlapping* binding sites, while Figure 6B shows the RBPs and microRNAs that have *neighboring* binding sites. Table 4 lists the details of the RBP-microRNA pairs that have the highest number of *overlapping* or *neighboring* binding sites. The complete list is in the Supplementary Table S11.

**TABLE 4:** List of RBP-microRNA pairs that have the highest number of *overlapping* and *neighboring* binding sites.

RBP	miRNA	Number of *overlapping* binding sites	Number of target genes with *overlapping* binding sites	Number of target genes with *neighboring* binding sites
TARDBP (TDP-43)	hsa-miR-181a-5p	499	499	613
TARDBP (TDP-43)	hsa-miR-181b-5p	499	499	613
TARDBP (TDP-43)	hsa-miR-181c-5p	499	499	613
TARDBP (TDP-43)	hsa-miR-181d-5p	499	499	613
ATXN2	hsa-miR-124-3p	455	455	1012
ATXN2	hsa-miR-325-3p	426	426	1019
CPSF7	hsa-miR-101-3p.2	340	340	435
ELAVL1	hsa-miR-340-5p	338	338	724
ATXN2	hsa-miR-340-5p	319	319	760
ATXN2	hsa-miR-181a-5p	318	318	767

*The first two columns are the names of the RBP and microRNA, the 3rd column is the number of *overlapping* binding sites between these two *trans* regulators, the 4th column is the number of mRNAs on which these regulators have *overlapping* binding sites, the 5th column is the number of mRNAs on which these two regulators have *neighboring* binding sites.*

For any pair of RBP and microRNA, there is 46.3% chance (average 42.2% in randomized simulation) that they have *overlapping* binding sites on at least one mRNA transcript, and 93.3% chance that they have *neighboring* binding sites on at least one mRNA. From Figure 6A and Table 4, we can see that several RBPs such as TARDBP (TDP-43), ATXN2, ELAVL1 (HuR) dominated among the RBP-miRNA pairs that had high number of overlapping binding sites. One potential reason is that these RBPs tend to have high number of binding sites and mRNA targets; indeed, the RBPs that have the highest number of binding sites also tend to have higher number of overlapping microRNA target sites (Pearson correlation 0.97, *p*-value = 1.36-E116). Also, since many microRNAs belong to the same family, they also have the same number of *overlapping* or *neighboring* binding sites with RBPs, e.g., hsa-miR-181a, hsa-miR-181b, hsa-miR-181c, hsa-miR-181d in Table 4.

TARDBP (TDP-43), the top RBP listed in Table 4, is known to bind to both DNA and RNA and can regulate RNA editing as well (Quinones-Valdez et al., 2019); it also interacts with Drosha and Dicer complexes and directly regulates the biogenesis of a subset of microRNAs (Kawahara and Mieda-Sato, 2012; Chen et al., 2018). A recent study has shown that TARDBP can bind to the 3′ UTR of pluripotency factors including Sox 2 and protect these transcripts from degradation mediated by microRNAs such as miR-21 (Modic et al., 2019). Interestingly, both TARDBP and ATXN2 are implicated in neurological disorders such as amyotrophic lateral sclerosis (ALS) (Sreedharan et al., 2008; Elden et al., 2010; Prasad et al., 2019; Yang et al., 2019); in addition, Ataxin-2 has been implicated in microRNA regulation (McCann et al., 2011; Ostrowski et al., 2017).

We next searched the ATtRACT database (Giudice et al., 2016), which has curated RBP binding motifs and positional weight matrices (PWM) determined from pull-down experiments or *in vitro* methods such as RNAcompete (Ray et al., 2013). For the RBPs shown in Table 4, we only found curated binding motifs for TARDBP (TDP-43) and ELAVL1 (HuR) in this database. TARDBP (TDP-43) has a canonical RNA Recognition Motif (RRM) and it has consensus binding motifs of 5′GAAUGG3′ or 5′GAAUGU3′ as determined by RNAcompete. Hsa-MiR-181 has a mature sequence of 5′AACAUUCAACGCUGUCGGUGAGU3′, which gives rise to seed region of 5′ACAUUCA3′. The complementary target site on the mRNA is 5′UGAAUGU3′, which strongly resembles the curated binding motifs of TARDBP. ELAVL1 (HuR) has consensus U-rich binding motifs. Hsa-miR-340-5p has a mature sequence of 5′UUAUAAAGCAAUGAGACUGAUU3′, which gives rise to seed region of 5′UAUAAAG3′. The complementary target site on the mRNA is 5′CUUUAUA″, which bears resemblance of the U-rich binding motifs of ELAVL1 (HuR). Therefore, the high prevalence of overlap between RBP-microRNA pairs can be explained by the similarity of their binding preferences.

### Regression Analysis Between *Trans* Regulators Based on TCGA Gene Expression Data

The above sections only investigated the potential of interactions between *trans* factors based on the location of their binding sites on the 3′ UTR of mRNAs. Next, we set out to analyze gene expression data to determine whether there are indeed interactions between these *overlapping* or *neighboring trans* regulators. The most definitive evidence to validate interactions between two *trans* factors is to experimentally remove one *trans* factor or its target site, then ascertain whether the regulatory effect of the other *trans* factor is elevated (competition) or diminished (cooperation). Such approaches were previously taken in the study of competitive effect between microRNAs (van Dongen et al., 2008; Khan et al., 2009), and between selected microRNA and RBPs (Kim et al., 2009; Glorian et al., 2011). Since the goal of our study is to conduct a global analysis and identify potential interactions between *trans* factors, we need an effective statistical method for this purpose. Toward this goal, we adopted a simplified statistical approach (Eq. 1), in which *x*_1_ and *x*_2_ represent the expression level of two *trans* regulators (RBPs or miRNAs), *a*_1_ represents the “regulatory strength” that *y* is regulated by *x*_1_, *a*_2_ represents the regulatory strength that *y* is regulated by *x*_2_, *a*_3_ is the interaction effect between *x*_1_ and *x*_2_, and *a*_4_ is the random error term, representing regulatory effect by other factors. If indeed there are interactions between two *trans* factors, either competitive or cooperative in a specific cancer type, we are likely to see statistically significant interactions between these two *trans* factors.

Equation 1:

(1) $$y=\,a_{1}\,x_{1}+a_{2}\,x_{2}+a_{3}\,x_{1}\,x_{2}+a_{4}$$

We downloaded the TCGA gene expression data from the Broad Institute FIREHOSE website (version 2016_01_28), and tested Eq. 1 on the following cancer types that have the greatest number of expression datasets: BRCA, THCA, LIHC, LUSC, LUAD, PRAD, KIRC. The number of gene expression datasets for these cancer types is listed in Supplementary Table S1.

For each cancer type, TCGA contains matched data for both cancer patients and normal controls. We conducted separate studies on three different gene expression datasets: tumor samples only, normal control samples only, and a union of tumor and normal control samples (results are summarized in Tables 5A–C, respectively). We first conducted regression analysis on RBP-microRNA pairs, i.e., *x*_1_ represents expression of an RBP, *x*_2_ represents expression level of a microRNA in Eq. 1. For each dataset and for each cancer type, we enumerated all the *Overlapping*, *Neighboring*, and *Independent* RBP-microRNA pairs, and conducted regression analysis on their expression profiles to identify interacting pairs. We used the R package p.adjust to calculate the *p*-values adjusted by Benjamini–Hochberg method (*q*-value < 0.05). If our hypothesis is correct, we expect to see more RBP-microRNA pairs that have statistically significant *p*-value on *a*_3_ term; in other words, more interacting pairs among *Overlapping* factors, fewer among *Neighboring* factors and the fewest among *Independent* factors. To test such hypothesis, we used Kolmogorov–Smirnov (K–S) test to compare between these three groups, i.e., the *Overlapping* pairs, the *Neighboring* pairs and the *Independent* pairs, to see which group has collectively lower *p*-values, i.e., which group has more pairs of interacting *trans* factors.

**TABLE 5:** Summary of regression analysis on RBP-microRNA pairs.

Cancer type	Comparing *overlapping* and *neighboring* pairs	Comparing *overlapping* and *independent* pairs	Comparing *neighboring* and *independent* pairs
**(A) Kolmogorov–Smirnov test in tumor samples**			
BRCA	0.97	0.96	0.62
KIRC	**1.88E-19**	**1.94E-15**	0.48
LIHC	**7.71E-14**	**1.68E-15**	0.42
LUAD	**3.04E-08**	**3.15E-108**	**0**
LUSC	0.27	**8.49E-34**	**0**
PRAD	0.10	**8.30E-231**	**0**
THCA	0.92	**4.20E-13**	**8.91E-254**

**(B) Kolmogorov–Smirnov test in normal samples**			

BRCA	**6.75E-04**	**7.28E-41**	**9.80E-253**
KIRC	0.90	0.99	1
LIHC	**2.73E-13**	**2.21E-34**	**3.87E-57**
LUAD	**7.39E-03**	1	0.99
LUSC	1.27E-02	0.86	0.99
PRAD	**7.01E-11**	**3.61E-09**	0.32
THCA	1.32E-03	**5.33E-27**	**1.22E-148**

**(C) Kolmogorov–Smirnov test in pooled tumor and normal samples**			

BRCA	0.80	0.94	1.00
KIRC	0.24	0.06	1.95E-02
LIHC	**9.01E-28**	**1.43E-61**	**6.52E-72**
LUAD	6.42E-06	**4.05E-108**	**0**
LUSC	7.79E-05	**6.38E-89**	**0**
PRAD	0.28	**4.34E-195**	**0**
THCA	0.22	**6.83E-26**	**1.85E-249**

**p*-value cutoff is chosen as 0.0008 after Bonferroni correction (0.05/63, since 63 tests are simultaneously conducted), significant *p*-values are shown in bold.*

Table 5A lists the analysis results from the tumor samples of the TCGA dataset. We found that, in most of the tumor types except for BRCA, collectively, the “*Overlapping*” pairs of RBP and microRNA had significantly lower *p*-values than “*Independent*” pairs (Column 3). It is likely that the breast cancer samples consist of subtypes and larger differences between tumor samples, which makes it less efficient in the regression analysis as outlined in Eq. 1. When comparing between “*Neighboring*” and “*Independent*” pairs (Column 4), in four cancer types (LUAD, LUSC, PRAD, THCA), *Neighboring* pairs have collectively lower *p*-values than *Independent* pairs, which is consistent with our expectations. In contrast, Column 2 shows that the *Overlapping* RBP-microRNA pairs have collectively lower *p*-values than *Neighboring* pairs only in three cancer types (KIRC, LIHC, LUAD). In our model (Figure 1A), both the *Overlapping* and *Neighboring* pairs can interact with each other, although in different mode. The *Overlapping* factors directly compete for the same target sequence in the mRNA transcript, while the *Neighboring* factors interact with each other in an indirect manner. Taken this altogether, the results in Table 5A is consistent with our hypothesis that *Overlapping* and *Neighboring* pairs of RBP and microRNA can interact with each other on regulating common gene targets.

Table 5B lists a similar comparison on the normal control samples from TCGA, while Table 5C lists the results from the pooled tumor and control samples, on pairs of RBPs and microRNAs. The results in Table 5C is mostly consistent with the results in Table 5A, while the results from normal control samples (Table 5B) reveals fewer interacting RBP-microRNA pairs as indicated by the modest *p*-values between *Overlapping* and *Independent* pairs, and between *Neighboring* and *Independent* pairs. It is not clear why the normal control samples are not as effective as cancer samples in revealing interacting RBP-microRNA pairs. One potential reason could be the relative homogeneity in gene expression among the normal control samples, which reduces the statistical power in the regression analysis (Eq. 1). We also note that such widespread interactions between RBPs and microRNAs are observed in some cancer types but not in others. It could be because the gene expression data from certain cancer types have limited statistical power, due to study design, or the homogeneity of the cohort. Regardless, as shown in Table 5A, we indeed see the definitive evidence of interacting *trans* acting pairs in the tumor samples.

Table 5 shows the results of regression between RBPs and microRNAs, we next conducted regression analysis between RBP-RBP pairs (Table 6) and between microRNA-microRNA pairs (Table 7). Consistent with our hypothesis, Table 6 shows that, collectively, there are significantly more regulatory interactions among *Overlapping* and *Neighboring* pairs of RBPs than *Independent* pairs (Column 3 and 4); the prevalence of interactions between *Overlapping* pairs and *Neighboring* pairs of RBPs are statistically indistinguishable (Column 2). Regression analysis of microRNA pairs from TCGA tumor samples are shown in Table 7, which shows *Overlapping* pairs collectively have more interactions than *Neighboring* and *Independent* pairs, and *Neighboring* pairs have more prevalent interactions than *Independent* pair.

**TABLE 6:** Summary of regression analysis on RBP-RBP pairs: Kolmogorov–Smirnov test in tumor samples.

Cancer type	Comparing *overlapping* and *neighboring* pairs	Comparing *overlapping* and *independent* pairs	Comparing *neighboring* and *independent* pairs
BRCA	1	0.06	**0**
KIRC	1	1	**0**
LIHC	1	**4.71E-151**	**0**
LUAD	0.96	**0**	**0**
LUSC	1	**0**	**0**
PRAD	1	**0**	**0**
THCA	1	1	**0**

**p*-value cutoff is chosen as 0.002 after Bonferroni correction (0.05/21, since 213 tests are simultaneously conducted), significant *p*-values are shown in bold.*

**TABLE 7:** Summary of regression analysis on microRNA-microRNA pairs: Kolmogorov–Smirnov test in tumor samples.

Cancer type	Comparing *overlapping* and *neighboring* pairs	Comparing *overlapping* and *independent* pairs	Comparing *neighboring* and *independent* pairs
BRCA	**1.24E-58**	**0**	**0**
KIRC	0.99	0.98	**0**
LIHC	**8.77E-37**	**9.32E-307**	0.42
LUAD	**3.13E-156**	**0**	**0**
LUSC	**2.30E-33**	**0**	**0**
PRAD	1	**4.19E-83**	**0**
THCA	**9.62E-292**	**0**	**0**

**p*-value cutoff is chosen as 0.002 after Bonferroni correction (0.05/21, since 213 tests are simultaneously conducted), significant *p*-values are shown in bold.*

In the following we focus our analysis to the interactions between RBP-microRNA pairs. We ask whether the statistically significant RBP-microRNA pairs and significant RBP-microRNA-mRNA trios that we observe in one cancer type are also found in other cancer types. If indeed this is the case, then it would be considered as additional evidence for the validity of such interactions. Indeed, many of such interacting pairs or trios are found in more than one cancer type. However, we need to remove one confounding factor, as many of these RBP-microRNA pairs are present more frequently than other pairs in the input data (Eq. 1). We conducted a hypergeometric Fisher’s exact test and calculated the *p*-value of these frequently occurring RBP-microRNA pairs in each cancer types. Supplementary Table S12 lists the top occurring RBP-microRNA pairs and their *p*-values (Fisher’s exact test), and Supplementary Table S13 lists the top significant RBP-microRNA-mRNA trios and their *p*-values and *q*-values after Benjamini–Hochberg correction. The complete list of significant RBP-microRNA pairs and RBP-microRNA-mRNA trios from each cancer type can be found in the Supplementary Tables S14, S15 (also see the Venn diagrams in Figure S3). The detail of RBP-RBP pairs and RBP-RBP-mRNA trios are in Supplementary Tables S16–S19 (also see the Venn diagram in Figure S4), and the detail of microRNA-microRNA pairs and microRNA-microRNA-mRNA trios are in Supplementary Tables S20–S23 (also see the Venn diagram in Supplementary Figure S5).

## Discussion

In this study, we analyzed 171 RBPs for which there are high quality CLIP-Seq experimental data available. This represents only a small fraction of the human RBP repertoire (424 known and predicted RBPs (Ray et al., 2013) or 1,542 manually curated RBPs (Gerstberger et al., 2014). We also used the predicted microRNA target sites instead of experimentally validated microRNA target sites, which is still scarce at the moment. Despite such limitations, we think our combined two-prong analysis on both gene expression and sequence data is still robust to discover regulatory interactions between these *trans* regulators. There are two lines of evidence to support this. Many of the *trans* interactions we discovered based on regression analysis were previously reported in the literature, such as ELAVL1 (HuR) and miR-19 (Kim et al., 2009; Glorian et al., 2011), F6 and YTHDC1 (Kasowitz et al., 2018). In addition, many of these interactions between *trans* factors are discovered in multiple cancer types, after controlling for false discovery rate.

Many of the identified *trans* regulators such as HuR have a large number of targets in the cell, so there is a possibility that some of these predicted or observed interactions between *trans* regulators are indirect effects instead of direct effects manifested on the same mRNA. The only definitive way to validate these *trans* regulatory effects is to quantify the individual regulatory effect of these *trans* factors and ascertain whether the combined regulatory effect is additive or not, similar to the study of epistasis or genetic interaction between regulatory elements. With the accumulation of genetic variation and individual gene expression data from projects such as GTEx, it may be feasible in the near future to achieve such a goal.

We showed evidence that some of these RBPs and microRNAs have cooperative or competitive effect in gene regulation; some of these *trans* regulators or target genes are involved in cancer or neurological disorders. A potentially intriguing and useful application of such *trans* co-regulation effect is to manipulate one *trans* regulator to enhance or attenuate the effect of another *trans* regulator. Several intriguing examples had been reported in the literature, for example knocking down a microRNA to sensitize cancer cell’s response to drugs that target RBPs (Gabra and Salmena, 2017). We hope our systematic analysis will provide some insight on understanding the interaction and mechanism of *trans* factors in human diseases and on designing on effective therapeutic approaches.

## Data Availability Statement

All datasets generated for this study are included in the article/Supplementary Material.

## Author Contributions

YL and ZZ conceived the study and wrote the manuscript. YL developed the algorithm and analyzed the results. DK, JL, and ZZ supervised the study. All authors have read and approved the final manuscript.

## Conflict of Interest

The authors declare that the research was conducted in the absence of any commercial or financial relationships that could be construed as a potential conflict of interest.

## Footnotes

1 http://lulab.life.tsinghua.edu.cn/

2 https://gdac.broadinstitute.org

## Supplementary Material

FIGURE S1 Percentage of RBP-RBP pair that have *neighboring* or *overlapping* target sites. Red circles represent the percentage of RBP-RBP pair with *neighboring* target sites, blue circles represent the percentage of RBP-RBP pair with *overlapping* target sites.

FIGURE S2 MicroRNA pairs that have overlapping or neighboring target sites. Color in each cell represents the number of genes on which the two microRNAs have *overlapping* (left) or *neighboring* (right) binding sites. microRNAs are listed in the same order on both *X*- and *Y*-axis.

FIGURE S3 Intersection of RBP-RBP-mRNA trios in seven types of tumors. **(A)** Intersection of *overlapping* trios among cancer types before filtering by regression analysis on expression profiles. **(B)** Intersection of *overlapping* trios after regression analysis as outlined in Eq. 1.

FIGURE S4 Intersection of microRNA-microRNA-mRNA trios in seven types of tumors. **(A)** Intersection of *overlapping* trios among cancer types before filtering by regression analysis on expression profiles. **(B)** Intersection of *overlapping* trios after regression analysis as outlined in Eq. 1.

FIGURE S5 Intersection of RBP-microRNA-mRNA trios in seven types of tumors. **(A)** Intersection of *overlapping* trios among cancer types before filtering by regression analysis on expression profiles. **(B)** Intersection of *overlapping* trios after regression analysis as outlined in Eq. 1. Some of these data tables are too big to upload, they can be found at the website: http://sites.utoronto.ca/zhanglab/papers/RBP_miR/.

TABLE S1 Summary of TCGA data. Number of patients for each cancer type. Each row represents a type of cancer. Column 3 and 4 represent the number of miRNA and mRNA after we remove the miRNA and mRNA with more than 30% missing value. Column 5 and 6 represent the number of normal samples and tumor samples.

TABLE S2 Summary of RBP binding data as collected by POSTAR2 database and filtered after considering structure accessibility.

TABLE S3 Binding sites of all the RBPs. (Please see website: http://sites.utoronto.ca/zhanglab/papers/RBP_miR/) Each row represents an RBP. The first column is the name of the RBP, the next block of five columns represents the chromosome number, coordinates, strand and the name of the target gene.

TABLE S4 RBP binding sites on genes. (Please see website: http://sites.utoronto.ca/zhanglab/papers/RBP_miR/). This table represents essentially the same information as Supplementary Table S3, but from the perspective of target genes. Each row represents a target gene. The first column is the name of the target gene; the next block of five columns represent the chromosome number, coordinates, name of RBP and the chromosomal strand.

TABLE S5 The number of RBP regulators per gene. This is the complete version of Table 2. Each row represents a gene; the rows are sorted in descending order according to the number of bound RBPs.

TABLE S6 Complete list of overlapping RBP binding sites. This is a complete version of Table 3. Each row represents an RBP-RBP pair, rows are sorted in descending order according to the number of overlapping binding sites.

TABLE S7 Binding sites of all the microRNAs sorted by microRNAs. (Please see website: http://sites.utoronto.ca/zhanglab/papers/RBP_miR/). Each row represents a microRNA; the chromosomal coordinates of the binding sites and the name of the gene targeted by the microRNA are listed as well.

TABLE S8 Binding sites of all the microRNAs sorted by target genes. (Please see website: http://sites.utoronto.ca/zhanglab/papers/RBP_miR/). Each row represents a target gene; the chromosomal coordinates of the binding sites and the name of the microRNA regulator are listed as well.

TABLE S9 List of microRNA-microRNA pairs that have the highest number of *overlapping* and *neighboring* binding sites. The first two columns are the names of the microRNAs, the 3rd column is the number of *overlapping* binding sites between these two microRNAs, the 4th column is the number of mRNAs on which these two microRNAs have *overlapping* binding sites.

TABLE S10 Complete list of overlapping microRNA-microRNA binding sites. This is a complete version of Supplementary Table S9. Each row represents a microRNA-microRNA pair, the rows are sorted in descending order according to the number of overlapping binding sites.

TABLE S11 Complete list of overlapping RBP-microRNA binding sites. This is a complete version of Table 4. Each row represents an RBP-microRNA pair, and these rows are sorted in descending order according to the number of overlapping binding sites.

TABLE S12 Top 100 RBP-microRNA pairs from regression analysis in 7 cancers. We use Fisher’s exact test to calculate the *p*-value of frequently occurring RBP-microRNA pairs in each cancer. Each row represents an RBP-microRNA pair. We sort the rows in ascending order according to *p*-value and select top 100 RBP-microRNA pairs for each cancer. Column 1 and 2 represent microRNA and RBP. Columns 3–9, respectively represent the *p*-value calculated by Fisher’s exact test in BRCA, KIRC, LIHC, LUAD, LUSC, PRAD, and THCA.

TABLE S13 Top 100 RBP-microRNA-mRNA trios form regression analysis in seven cancers. Top RBP-microRNA-mRNA trios. We used regression analysis to calculate the significant *p*-value to RBP-microRNA-mRNA trios and used Benjamini–Hochberg method to adjust the *p*-value to get the *q*-value. Each row represents an RBP-microRNA-mRNA trio. We sort the rows in ascending order according to *q*-value and select top 100 RBP-microRNA-mRNA trios for each cancer. Column 1, 2, and 3 represent microRNA, RBP, and mRNA. Column 4, 5, 6, 7, 8, 9, and 10, respectively represent *p*-value/*q*-value calculated in BRCA, KIRC, LIHC, LUAD, LUSC, PRAD, and THCA. Column 11 represents the number of overlapping binding sites in RBP-microRNA-mRNA trios. Column 12 represents the ratio between overlapping binding sites and the sum of the number of microRNA target sites and RBP binding sites.

TABLE S14 Complete list of significant RBP-microRNA pairs in seven cancers. We used *q*-value less than 0.05 to screen the RBP-microRNA pairs for each cancer and count the number of their occurrences in multiple cancers. Column 1 and 2 represent microRNA and RBP. Column 3, 4, 5, 6, 7, 8, and 9, respectively represent the *p*-value calculated by Fisher’s exact test in BRCA, KIRC, LIHC, LUAD, LUSC, PRAD, and THCA. Column 10 represents the number of the pair occurrences in multiple cancers. We sorted these rows in descending order according to column 10.

TABLE S15 Complete list of significant RBP-microRNA-mRNA trios in seven cancers. (Please see website: http://sites.utoronto.ca/zhanglab/papers/RBP_miR/). We used *q*-value less than 0.05 to screen the RBP-microRNA-mRNA trios for each cancer and count the number of their occurrences in multiple cancers. Column 1, 2 and 3 represent microRNA, RBP, and mRNA. Column 4, 5, 6, 7, 8, 9, and 10, respectively represent the *p*-value/*q*-value calculated in BRCA, KIRC, LIHC, LUAD, LUSC, PRAD, and THCA. Column 11 represents the number of the trio occurrences in multiple cancers. We sorted these rows in descending order according to column 11.

TABLES S16–S19 are for RBP-RBP pairs and RBP-RBP-mRNA trios.

TABLE S16 Top 100 significant RBP-RBP pairs in seven cancers.

TABLE S17 Top 100 significant RBP-RBP-mRNA trios in seven cancers.

TABLE S18 Complete list of significant RBP-RBP pairs in seven cancers.

TABLE S19 Complete list of significant RBP-RBP-mRNA trios in seven cancers. (Please see website: http://sites.utoronto.ca/zhanglab/papers/RBP_miR/).

TABLES S20–S23 are for microRNA-microRNA pairs and microRNA-microRNA-mRNA trios.

TABLE S20 Top 100 significant microRNA-microRNA pairs.

TABLE S21 Top significant microRNA-microRNA-mRNA trios.

TABLE S22 Complete list of significant microRNA-microRNA pairs.

TABLE S23 Complete list of significant microRNA-microRNA-mRNA trios.

TABLE S24 Summary of regression results. **(A)** The summary of regression results on RBP-RBP-mRNA. Each row represents a type of cancer. Column 2, 3, and 4, respectively represent the number of RBP, RBP, mRNA which show in our processed TCGA tumor samples. Column 5 represents the number of RBP-RBP-mRNA trios with overlapping binding sites. Column 6 represents the number of RBP-RBP-mRNA trios with overlapping binding sites screened by regression analysis. **(B)** The summary of regression results on microRNA-microRNA-mRNA. Each row represents a type of cancer. Column 2, 3, and 4, respectively represent the number of microRNA, microRNA, mRNA which show in our processed TCGA tumor samples. Column 5 represents the number of microRNA-microRNA-mRNA trios with overlapping binding sites. Column 6 represents the number of microRNA-microRNA-mRNA trios with overlapping binding sites screened by regression analysis. **(C)** The summary of regression results on RBP-microRNA-mRNA. Each row represents a type of cancer. Column 2, 3, and 4, respectively represent the number of RBP, microRNA, mRNA which show in our processed TCGA tumor samples. Column 5 represents the number of RBP-microRNA-mRNA trios with overlapping binding sites. Column 6 represents the number of RBP-microRNA-mRNA trios with overlapping binding sites screened by regression analysis.
