# Supplementary material for: A Survey of Regulatory Interactions Among RNA Binding Proteins and MicroRNAs in Cancer
Source: Front Genet. 2020 Sep 8;11:515094. doi: 10.3389/fgene.2020.515094 (PMC7506142; doi:10.3389/fgene.2020.515094)
Supplement: FIGURE S1 — Percentage of RBP-RBP pair that have neighboring or overlapping target sites. Red circles represent the percentage of RBP-RBP pair with neighboring target sites, blue circles represent the percentage of RBP-RBP pair with overlapping target sites. [file Data_Sheet_1.PDF]

Supplementary Material

1 Supplementary Data

Please see Section Supplementary Tables.

2 Supplementary Figures and Tables

2.1 Supplementary Figures

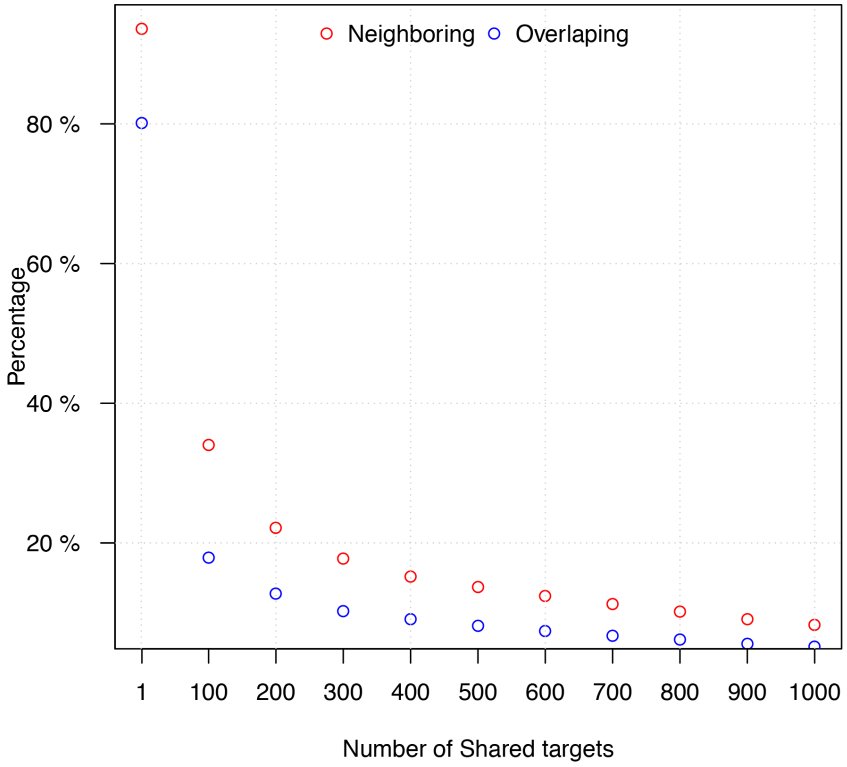

Supplementary Figure S1. Percentage of RBP-RBP pair that have *neighboring* or *overlapping* target sites. Red circles represent the percentage of RBP-RBP pair with *neighboring* target sites, blue circles represent the percentage of RBP-RBP pair with *overlapping* target sites.

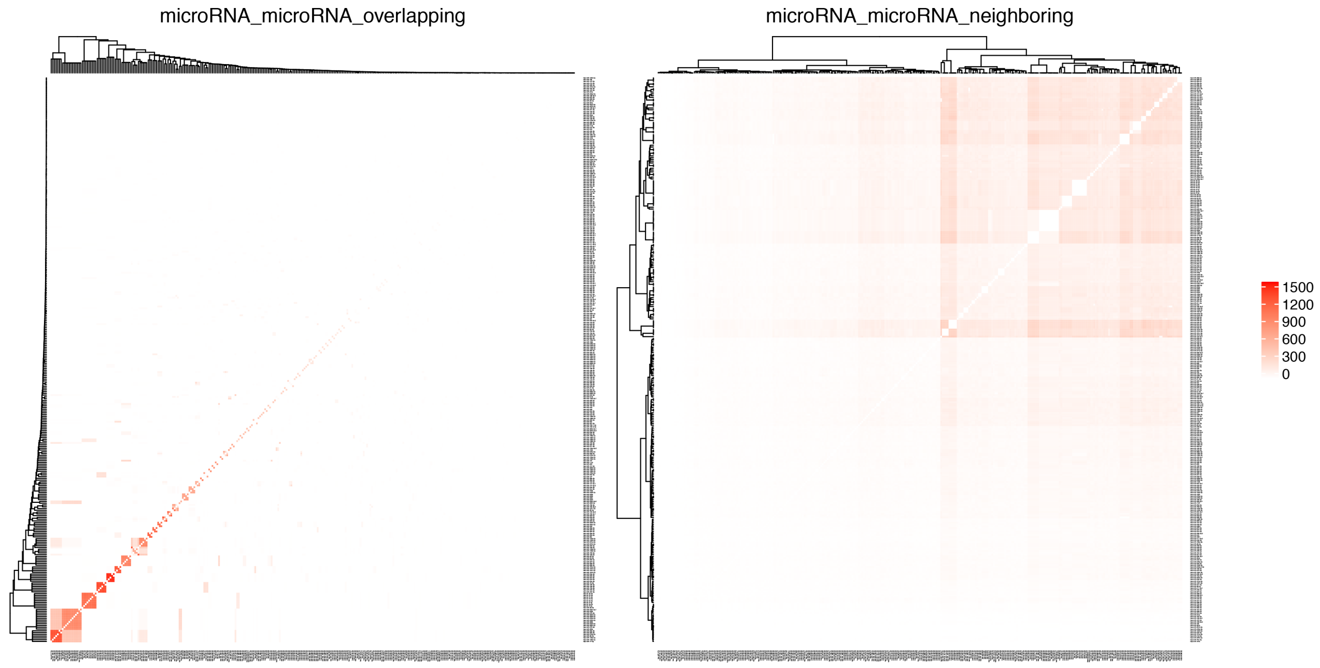

Figure S2. microRNA pairs that have overlapping or neighboring target sites. Color in each cell represents the number of genes on which the two microRNAs have *overlapping* (left) or *neighboring* (right) binding sites. microRNAs are listed in the same order on both X and Y axis.

A. All overlapping RBP–RBP–mRNA trios

B. Significant overlapping RBP–RBP–mRNA trios

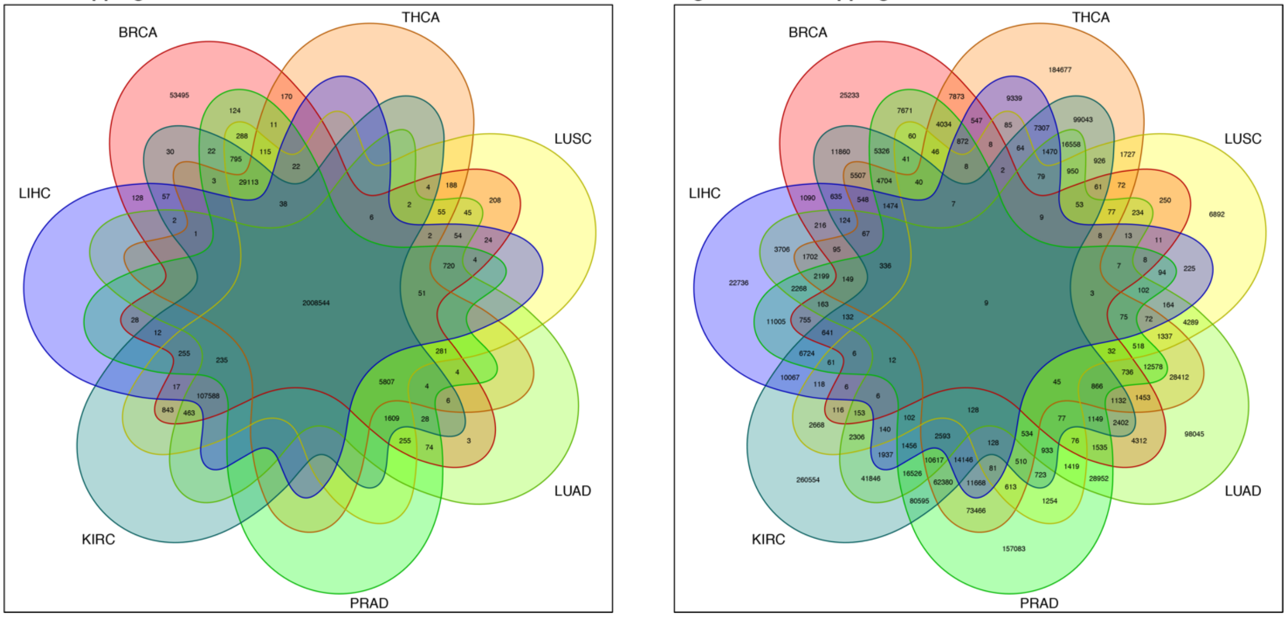

Supplementary Figure S3. Intersection of RBP-RBP-mRNA trios in 7 types of tumors. (A) Intersection of *overlapping* trios among cancer types before filtering by regression analysis on expression profiles. (B). Intersection of *overlapping* trios after regression analysis as outlined in Equation 1.

A. All overlapping microRNA–microRNA–mRNA trios

B. Significant overlapping microRNA–microRNA–mRNA trios

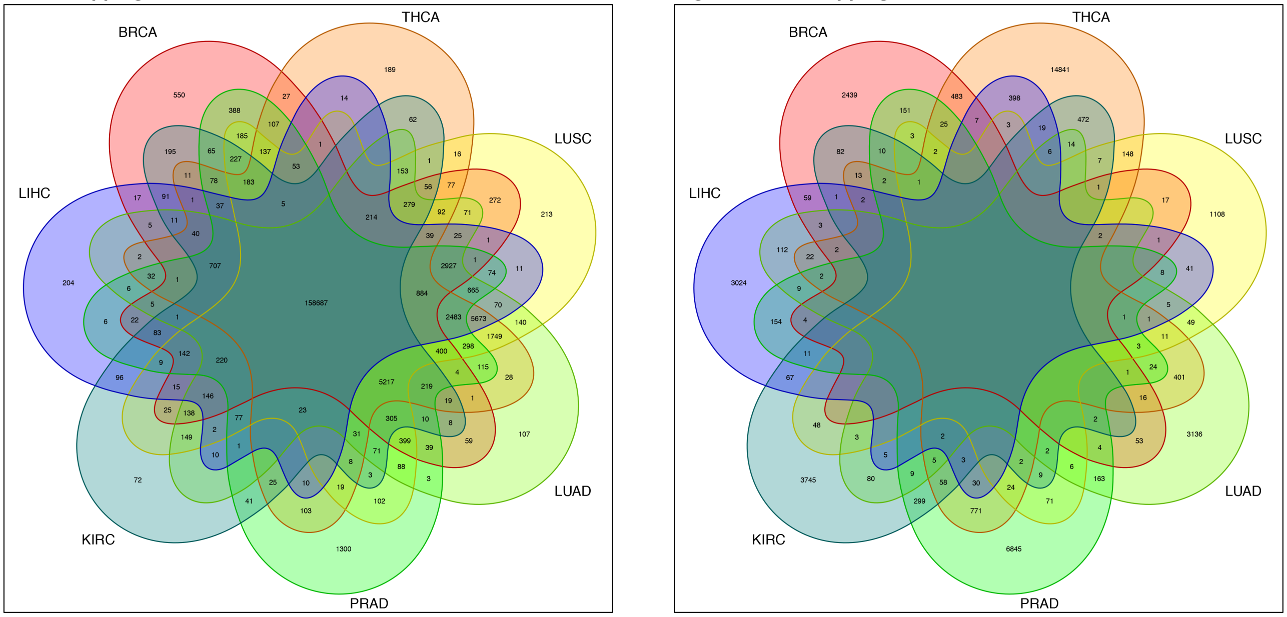

Figure S4. Intersection of microRNA-microRNA-mRNA trios in 7 types of tumors. (A) Intersection of *overlapping* trios among cancer types before filtering by regression analysis on expression profiles. (B). Intersection of *overlapping* trios after regression analysis as outlined in Equation 1.

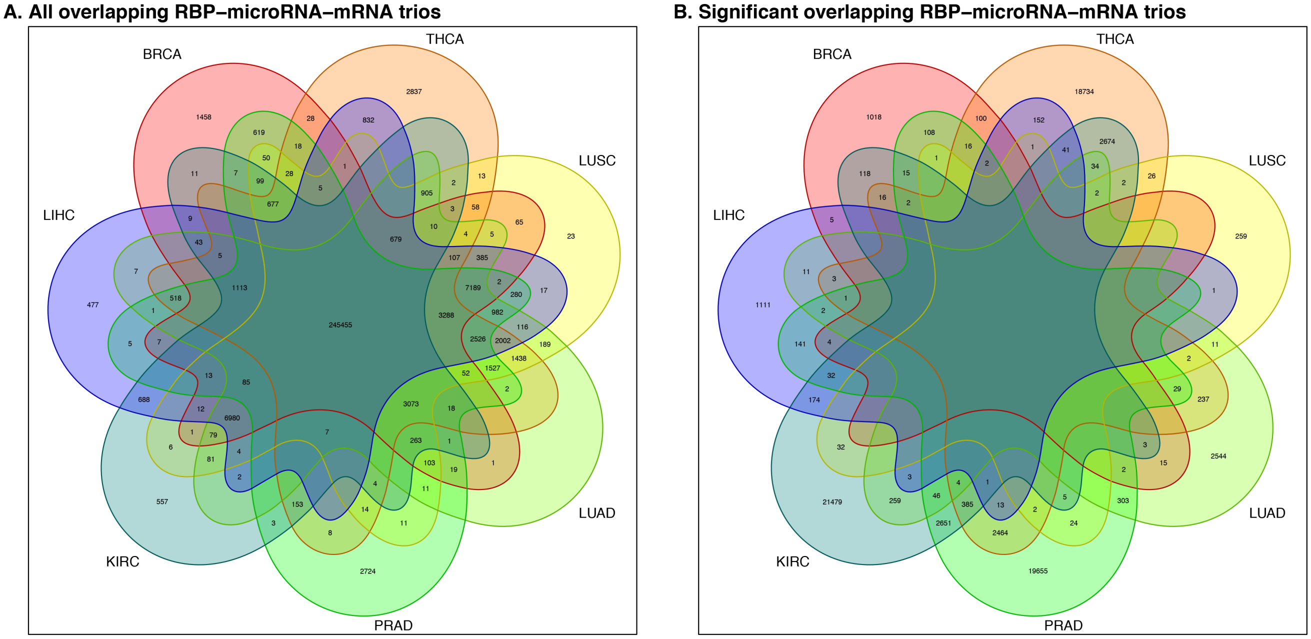

Figure S5. Intersection of RBP-microRNA-mRNA trios in 7 types of tumors. (A) Intersection of *overlapping* trios among cancer types before filtering by regression analysis on expression profiles. (B). Intersection of *overlapping* trios after regression analysis as outlined in Equation 1.

2.2 Supplementary Tables

Some of these data tables are too big to upload, they can be found at the website: http://sites.utoronto.ca/zhanglab/papers/RBP_miR/

Supplementary Table S1. summary of TCGA data
Number of patients for each cancer type. Each row represents a type of cancer. Column 3 and 4 represent the number of miRNA and mRNA after we remove the miRNA and mRNA with more than 30% missing value. Column 5 and 6 represent the number of normal samples and tumor samples.

Supplementary Table S2:
Summary of RBP binding data as collected by POSTAR2 database and filtered after considering structure accessibility.

Supplementary Table S3. Binding sites of all the RBPs.
(please see website: http://sites.utoronto.ca/zhanglab/papers/RBP_miR/)
Each row represents an RBP. The first column is the name of the RBP, the next block of five columns represents the chromosome number, coordinates, strand and the name of the target gene.

Supplementary Table S4. RBP binding sites on genes.
(please see website: http://sites.utoronto.ca/zhanglab/papers/RBP_miR/)
This table represents essentially the same information as Table S3, but from the perspective of target genes. Each row represents a target gene. The first column is the name of the target gene; the next block of five columns represent the chromosome number, coordinates, name of RBP and the chromosomal strand.

Supplementary Table S5. The number of RBP regulators per gene.
This is the complete version of Table 2. Each row represents a gene; the rows are sorted in descending order according to the number of bound RBPs.

Supplementary Table S6. Complete list of overlapping RBP binding sites.
This is a complete version of Table 3. Each row represents an RBP-RBP pair, rows are sorted in descending order according to the number of overlapping binding sites.

Supplementary Table S7. Binding sites of all the microRNAs sorted by microRNAs.
(please see website: http://sites.utoronto.ca/zhanglab/papers/RBP_miR/)
Each row represents a microRNA; the chromosomal coordinates of the binding sites and the name of the gene targeted by the microRNA are listed as well.

Supplementary Table S8. Binding sites of all the microRNAs sorted by target genes.
(please see website: http://sites.utoronto.ca/zhanglab/papers/RBP_miR/)
Each row represents a target gene; the chromosomal coordinates of the binding sites and the name of the microRNA regulator are listed as well.

Supplementary Table S9: List of microRNA-microRNA pairs that have the highest number of *overlapping* and *neighboring* binding sites. The first two columns are the names of the microRNAs, the 3rd column is the number of *overlapping* binding sites between these two microRNAs, the 4th column is the number of mRNAs on which these two microRNAs have *overlapping* binding sites.

Supplementary Table S10. Complete list of overlapping microRNA-microRNA binding sites.
This is a complete version of Supplementary Table S9. Each row represents a microRNA-microRNA pair, the rows are sorted in descending order according to the number of overlapping binding sites.

Supplementary Table S11. Complete list of overlapping RBP-microRNA binding sites**.**

This is a complete version of Table 4. Each row represents an RBP-microRNA pair, and these rows are sorted in descending order according to the number of overlapping binding sites.

Supplementary Table S12. Top 100 RBP-microRNA pairs from regression analysis in 7 cancers. We use Fisher's exact test to calculate the *p*-value of frequently occurring RBP-microRNA pairs in each cancer. Each row represents an RBP-microRNA pair. We sort the rows in ascending order according to *p*-value and select top 100 RBP-microRNA pairs for each cancer. Column 1 and 2 represent microRNA and RBP. Columns 3-9 respectively represent the *p*-value calculated by Fisher's exact test in BRCA, KIRC, LIHC, LUAD, LUSC, PRAD,THCA.

Supplementary Table S13. Top 100 RBP-microRNA-mRNA trios form regression analysis in 7 cancers.
Top RBP-microRNA-mRNA trios. We used regression analysis to calculate the significant *p*-value to RBP-microRNA-mRNA trios and used Benjamini-Hochberg method to adjust the *p*-value to get the q-value. Each row represents an RBP-microRNA-mRNA trio. We sort the rows in ascending order according to q-value and select top 100 RBP-microRNA-mRNA trios for each cancer. Column 1,2,and 3 represent microRNA, RBP and mRNA. Column 4,5,6,7,8,9,10 respectively represent *p*-value/q-value calculated in BRCA, KIRC, LIHC, LUAD, LUSC, PRAD, THCA. Column 11 represents the number of overlapping binding sites in RBP-microRNA-mRNA trios. Column 12 represents the ratio between overlapping binding sites and the sum of the number of microRNA target sites and RBP binding sites.

Supplementary Table S14. Complete list of significant RBP-microRNA pairs in 7 cancers.
We used q-value less than 0.05 to screen the RBP-microRNA pairs for each cancer and count the number of their occurrences in multiple cancers. Column 1 and 2 represent microRNA and RBP. Column 3,4,5,6,7,8,9 respectively represent the *p*-value calculated by Fisher's exact test in BRCA, KIRC, LIHC, LUAD, LUSC, PRAD,THCA. Column 10 represents the number of the pair occurrences in multiple cancers. We sorted these rows in descending order according to column 10.

Supplementary Table S15. Complete list of significant RBP-microRNA-mRNA trios in 7 cancers. (please see website: http://sites.utoronto.ca/zhanglab/papers/RBP_miR/)
We used q-value less than 0.05 to screen the RBP-microRNA-mRNA trios for each cancer and count the number of their occurrences in multiple cancers. Column 1, 2 and 3 represent microRNA, RBP and mRNA. Column 4,5,6,7,8,9,10 respectively represent the *p*-value/q-value calculated in BRCA, KIRC, LIHC, LUAD, LUSC, PRAD, THCA. Column 11 represents the number of the trio occurrences in multiple cancers. We sorted these rows in descending order according to column 11.

Supplementary Table S16-S19 are for RBP-RBP pairs and RBP-RBP-mRNA trios

Supplementary Table S16. Top 100 significant RBP-RBP pairs in 7 cancers

Supplementary Table S17. Top 100 significant RBP-RBP-mRNA trios in 7 cancers

Supplementary Table S18. Complete list of significant RBP-RBP pairs in 7 cancers

Supplementary Table S19. Complete list of significant RBP-RBP-mRNA trios in 7 cancers. (please see website: http://sites.utoronto.ca/zhanglab/papers/RBP_miR/)

Supplementary Table S20-S23 are for microRNA-microRNA pairs and microRNA-microRNA-mRNA trios

Supplementary Table S20. Top 100 significant microRNA-microRNA pairs.

Supplementary Table S21. Top significant microRNA-microRNA-mRNA trios.

Supplementary Table S22. Complete list of significant microRNA-microRNA pairs.

Supplementary Table S23. Complete list of significant microRNA-microRNA-mRNA trios

Supplementary Table S24: Summary of regression results
(A). The summary of regression results on RBP-RBP-mRNA. Each row represents a type of cancer. Column 2, 3 and 4 respectively represent the number of RBP,RBP, mRNA which show in our processed TCGA tumor samples. Column 5 represents the number of RBP-RBP-mRNA trios with overlapping binding sites. Column 6 represents the number of RBP-RBP-mRNA trios with overlapping binding sites screened by regression analysis.

(B). The summary of regression results on microRNA-microRNA-mRNA. Each row represents a type of cancer. Column 2, 3 and 4 respectively represent the number of microRNA, microRNA, mRNA which show in our processed TCGA tumor samples. Column 5 represents the number of microRNA-microRNA-mRNA trios with overlapping binding sites. Column 6 represents the number of microRNA-microRNA-mRNA trios with overlapping binding sites screened by regression analysis.

(C). The summary of regression results on RBP-microRNA-mRNA. Each row represents a type of cancer. Column 2, 3 and 4 respectively represent the number of RBP, microRNA, mRNA which show in our processed TCGA tumor samples. Column 5 represents the number of RBP-microRNA-mRNA trios with overlapping binding sites. Column 6 represents the number of RBP-microRNA-mRNA trios with overlapping binding sites screened by regression analysis.
