# Supplementary material for: A Survey of Regulatory Interactions Among RNA Binding Proteins and MicroRNAs in Cancer
Source: Front Genet. 2020 Sep 8;11:515094. doi: 10.3389/fgene.2020.515094 (PMC7506142; doi:10.3389/fgene.2020.515094)
Supplement: TABLE S1 — Summary of TCGA data. Number of patients for each cancer type. Each row represents a type of cancer. Column 3 and 4 represent the number of miRNA and mRNA after we remove the miRNA and mRNA with more than 30% missing value. Column 5 and 6 represent the number of normal samples and tumor samples. [file Data_Sheet_2.ZIP › Supplementary Table S3_RBP_binding_sites_all_empty.rtf]

Data table too big to upload, please see the website:http://sites.utoronto.ca/zhanglab/papers/RBP_miR/
