# Supplementary material for: A Survey of Regulatory Interactions Among RNA Binding Proteins and MicroRNAs in Cancer
Source: Front Genet. 2020 Sep 8;11:515094. doi: 10.3389/fgene.2020.515094 (PMC7506142; doi:10.3389/fgene.2020.515094)
Supplement: TABLE S1 — Summary of TCGA data. Number of patients for each cancer type. Each row represents a type of cancer. Column 3 and 4 represent the number of miRNA and mRNA after we remove the miRNA and mRNA with more than 30% missing value. Column 5 and 6 represent the number of normal samples and tumor samples. [file Data_Sheet_2.ZIP › Supplementary Table S9_miRNA-miRNA_top_overlap_pairs.docx]

**Supplementary Table S9.** List of microRNA-microRNA pairs that have the highest number of *overlapping* and *neighboring* binding sites. The first two columns are the names of the microRNAs, the 3^rd^ column is the number of *overlapping* binding sites between these two microRNAs, the 4^th^ column is the number of mRNAs on which these two microRNAs have *overlapping* binding sites.

| **miRNA 1** | **miRNA 2** | **Number of overlapping binding sites** | **Number of target genes with *overlapping* binding sites** |
| --- | --- | --- | --- |
| hsa-miR-15a-5p | hsa-miR-15b-5p | 1975 | 1314 |
| hsa-miR-15a-5p | hsa-miR-424-5p | 1975 | 1314 |
| hsa-miR-15a-5p | hsa-miR-497-5p | 1975 | 1314 |
| hsa-miR-15b-5p | hsa-miR-424-5p | 1975 | 1314 |
| hsa-miR-15b-5p | hsa-miR-497-5p | 1975 | 1314 |
| hsa-miR-424-5p | hsa-miR-497-5p | 1975 | 1314 |
| hsa-miR-15a-5p | hsa-miR-16-5p | 1974 | 1313 |
| hsa-miR-15a-5p | hsa-miR-195-5p | 1974 | 1313 |
| hsa-miR-15b-5p | hsa-miR-16-5p | 1974 | 1313 |
| hsa-miR-15b-5p | hsa-miR-195-5p | 1974 | 1313 |
