# Supplementary material for: A Survey of Regulatory Interactions Among RNA Binding Proteins and MicroRNAs in Cancer
Source: Front Genet. 2020 Sep 8;11:515094. doi: 10.3389/fgene.2020.515094 (PMC7506142; doi:10.3389/fgene.2020.515094)
Supplement: TABLE S1 — Summary of TCGA data. Number of patients for each cancer type. Each row represents a type of cancer. Column 3 and 4 represent the number of miRNA and mRNA after we remove the miRNA and mRNA with more than 30% missing value. Column 5 and 6 represent the number of normal samples and tumor samples. [file Data_Sheet_2.ZIP › Supplementary Table S2_POSTAR2_summary.docx]

**Supplementary Table S2:** Summary of RBP binding data as collected by POSTAR2 database and filtered after considering structure accessibility.

| Number of entries | 2,865,656 |
| --- | --- |
| Number of unique RBPs | 171 |
| Number of unique human mRNAs | 14,520 |
| Average number of binding sites per RBP | 16,758 |
| Average number of genes targeted by an RBP | 2,077 |
